# The Dynamics of Incomplete Lineage Sorting across the Ancient Adaptive Radiation of Neoavian Birds

**DOI:** 10.1371/journal.pbio.1002224

**Published:** 2015-08-18

**Authors:** Alexander Suh, Linnéa Smeds, Hans Ellegren

**Affiliations:** Department of Evolutionary Biology, Evolutionary Biology Centre (EBC), Uppsala University, Uppsala, Sweden; Massey University, NEW ZEALAND

## Abstract

The diversification of neoavian birds is one of the most rapid adaptive radiations of extant organisms. Recent whole-genome sequence analyses have much improved the resolution of the neoavian radiation and suggest concurrence with the Cretaceous-Paleogene (K-Pg) boundary, yet the causes of the remaining genome-level irresolvabilities appear unclear. Here we show that genome-level analyses of 2,118 retrotransposon presence/absence markers converge at a largely consistent Neoaves phylogeny and detect a highly differential temporal prevalence of incomplete lineage sorting (ILS), i.e., the persistence of ancestral genetic variation as polymorphisms during speciation events. We found that ILS-derived incongruences are spread over the genome and involve 35% and 34% of the analyzed loci on the autosomes and the Z chromosome, respectively. Surprisingly, Neoaves diversification comprises three adaptive radiations, an initial near-K-Pg super-radiation with highly discordant phylogenetic signals from near-simultaneous speciation events, followed by two post-K-Pg radiations of core landbirds and core waterbirds with much less pronounced ILS. We provide evidence that, given the extreme level of up to 100% ILS per branch in super-radiations, particularly rapid speciation events may neither resemble a fully bifurcating tree nor are they resolvable as such. As a consequence, their complex demographic history is more accurately represented as local networks within a species tree.

## Abstract 2

A study of ancient genetic variation reveals genomic evidence for near-simultaneous speciation at the base of Neoaves (a group containing most modern birds), which temporally coincides with the mass extinction of nonavian dinosaurs and archaic birds.

## Author Summary

The rise of modern birds began after the mass extinction of nonavian dinosaurs and archaic birds at the Cretaceous-Paleogene (K-Pg) boundary, about 66 million years ago. This coincides with the super-rapid adaptive radiation of Neoaves (a group that contains most modern birds), which has been difficult to resolve even with whole genome sequences. We reconstructed the genealogical fates of thousands of rare genomic changes (insertions of selfish mobile elements called retrotransposons), a third of which were found to be affected by a phenomenon known as incomplete lineage sorting (ILS), namely a persistence of polymorphisms across multiple successive speciation events. Astoundingly, we found that near the K-Pg boundary, speciation events were accompanied by extreme levels of ILS, suggesting a near-simultaneous, star-like diversification process that appears plausible in the context of instantaneous niche availability that must have followed the K-Pg mass extinction. Our genome-scale results provide a population genomic explanation as to why some species radiations may be more complex than a fully bifurcating tree of life. We suggest that, under such circumstances, ILS bears witness to the biological limitation of phylogenetic resolution.

## Introduction

The rich biodiversity of many organismal groups is the result of bursts of rapid species diversifications, with extreme examples in angiosperms [1] and vertebrates [2]. Among the latter, birds are one of the most speciose groups with a total of >10,500 recognized species that are proposed to be the result of mostly recent accelerations of diversification rates [3]. Nevertheless, the deep roots of 95% of these species lie within the ancient adaptive radiation of Neoaves, comprising all contemporary avian lineages except Palaeognathae (ratites and tinamous) and the Galloanserae (chicken and ducks). This massive radiation exhibits the highest known diversification rate among deep vertebrate radiations [2], coincides with the Cretaceous-Paleogene (K-Pg) boundary, and gave rise to 36 extant bird lineages within <15 million years (MY) [4]. Simulations suggest that the distribution of neoavian internode lengths causes a very high probability of gene tree–species tree incongruences [5], i.e., hemiplasy derived from incomplete lineage sorting (ILS) [6]. ILS denotes the persistence of ancestral polymorphisms across multiple successive speciation events and is followed by stochastic allele fixation in each descendant lineage, potentially making phylogenetic inference at the level of individual loci problematic.

Past studies on the extent of ILS during speciation have been restricted to recent divergences because homoplasy needs to be low. For example, divergences among great apes show that ~30% of the gorilla genome exhibits nucleotide substitution patterns incongruent with the human/chimpanzee/gorilla species tree [7]. In contrast, the characteristics of ILS remain to be explored in adaptive radiations. As virtually homoplasy-free phylogenetic rare genomic changes [8,9], retrotransposed elements (REs) exhibit conflicting phylogenetic signals only when their insertions occurred on short internodes; they can thus be used to localize and quantify ILS even on very deep timescales [8,10–13].

## Results and Discussion

We analyzed ~130,000 long terminal repeat (LTR) retrotransposons in the 48 recently sequenced bird genomes [4] and obtained 2,118 presence/absence patterns of insertions that occurred within the neoavian radiation and are distributed genome-wide (S1 Table, S1 Fig, S1 Data). These RE markers were obtained after visual inspection under strict criteria for coding of character states at orthologous RE loci (see Materials and Methods), because we aimed to minimize the two sources of potential homoplasy; independent RE insertion and precise excision.

### Two Thousand RE Markers with Minimal Homoplasy

Homoplasy via independent RE insertion requires the retrotransposition of the same RE subtype into precisely the same genomic location, in the same orientation, and featuring an identical target site duplication. In addition to these factors that make independent insertions very rare, the LTR retrotransposons studied here have a low copy number (e.g., 3,138 copies in the zebra finch genome), were active only for a short time period around the neoavian radiation [10], and show no target site preference among thousands of reconstructed ancestral target sequences of inserted elements (S2 Fig). We therefore propose that the probability of homoplasy caused by independent insertions among our RE markers is extremely low. Homoplasy via precise excision is the deletion of the RE insertion and one copy of the duplicated target site, but not a single bp more or less than that. These requirements make the occurrence of precise excisions very rare and we therefore visually inspected all of our markers for precise boundaries of presence/absence states and coded imprecise or poorly aligned boundaries as missing data. Altogether, we suggest that our 2,118 RE markers contain negligible homoplasy, and conflicts are instead due to ILS-derived hemiplasy.

To verify that incongruences constitute ILS-derived hemiplasy, Hormozdiari et al. [14] proposed to test for topological consistence between each RE marker and a sequence tree derived from its flanking nucleotides. However, we note that failure of this test for some of their RE markers does not equal homoplasy of RE markers. Alternative and more plausible causes for inconsistencies are homoplasy or tree reconstruction uncertainties in the flanking sequence trees and the fact that recombination may cause different topologies between adjacent loci [15]. Unfortunately, single-locus sequence trees of Neoaves have an average topological distance of 63% for introns and 66% for ultraconserved elements (UCEs) from the main Jarvis et al. tree [4]. This means that the average nonexonic locus fails to congruently resolve most of the neoavian internodes. We note that it is therefore not possible to independently verify hemiplasy in neoavian RE markers by comparison to flanking sequence trees. Nevertheless, if homoplasy was prevalent in our RE markers, we would expect to see an equal distribution of RE incongruences across all of the sampled clades of Neoaves. While we find dozens of presence/absence markers with incongruences affecting the short branches within the neoavian radiation (S1 Table; e.g., the core landbirds and core waterbirds clades), there is not a single RE incongruence in our presence/absence matrix (S1 Table) affecting well-accepted internal relationships within postradiation taxa, such as passerines, parrots, eagles, penguins, the woodpecker/bee-eater clade, the hummingbird/swift clade, and the flamingo/grebe clade. Such an imbalance of RE incongruences strongly implies that homoplasy is indeed negligible among our 2,118 RE markers.

### Genome-Scale RE-Based Phylogeny of Neoaves

**Fig 1:**
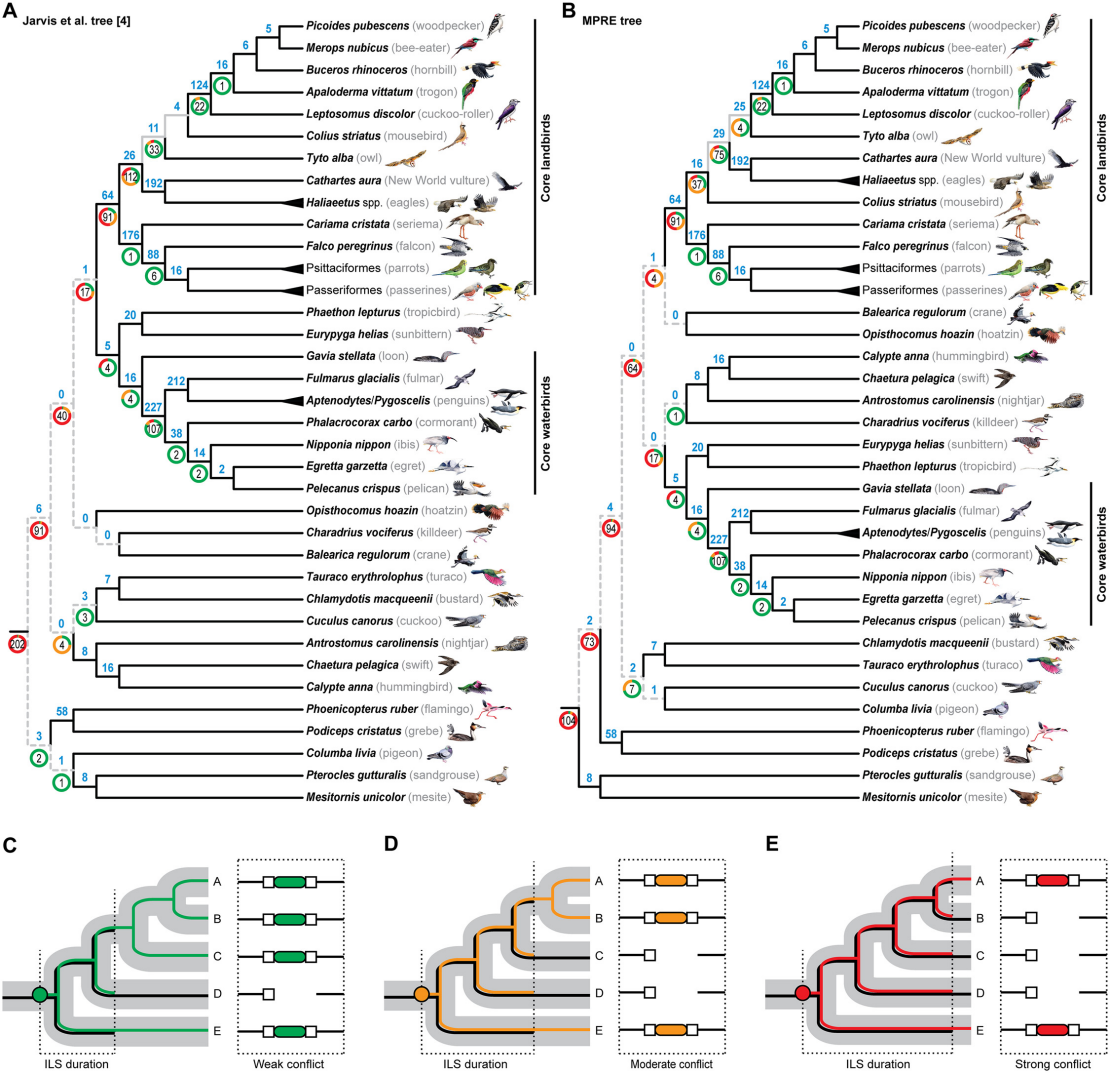
Phylogenetic tree of rare genomic changes reveals varying degree of incomplete lineage sorting across Neoaves diversification. (A) The main whole-genome sequence tree from Jarvis et al. [4] mapped with our 2,118 retrotransposon markers (745 incongruent markers; tree length = 5,579; consistency index = 0.40; retention index = 0.64). (B) The same markers mapped on the single MPRE tree (S2 Data) resulting from analysis of their 2,118 presence/absence patterns (720 incongruent markers; tree length = 5,377; consistency index = 0.41; retention index = 0.66) under Felsenstein’s polymorphism parsimony [16]. Black branches indicate topological concordances between the MPRE tree and the main Jarvis et al. tree [4], and discordances are limited to the deepest neoavian internodes (grey dashed branches) and the conflicting position of the mousebird (grey branches). The amount of ILS-free, conflict-free insertion events (blue bold numbers) was identified for each internode, and numbers within doughnut plots indicate counts of ILS-affected RE insertion events leading to the persistence of insertion polymorphisms across two (green), three (orange), or more (red parts of doughnut plots) speciation events. (C–E) Schematic illustration of the different genealogical fates of segregating presence (colored lines) or absence (black lines) alleles following RE insertion (colored circles) in an exemplary five-taxon species tree. We show one respective example for the different degrees of gene tree–species tree conflict that can be caused by incomplete lineage sorting (ILS) across two (C), three (D), or more than three (E) successive speciation events. Incongruence of RE presence/absence patterns (dashed boxes) is illustrated with REs as colored ovals, target site duplications as white squares, and orthologous genomic flanks as black lines. The bird paintings were generated by Jon Fjeldså (used with permission).

We analyzed the RE presence/absence matrix using Felsenstein’s polymorphism parsimony [16] and obtained a single most parsimonious RE (MPRE) tree, whose branches are supported by a total of 1,373 conflict-free insertion events across the neoavian radiation (Fig 1B). The topology is very similar to previous phylogenomic estimates using mostly noncoding nucleotide data [4,10,17–21], including relationships previously strongly supported in whole-genome sequence analyses [4] (Fig 1A), such as the sunbittern/tropicbird, bustard/turaco, and mesite/sandgrouse clades. From these three groups, only the sunbittern/tropic clade was previously recovered in some multilocus analyses [19–21].

The remaining 745 retrotransposon markers show different degrees of gene tree–species tree incongruence. This is best explained by the persistence of ancestral polymorphisms across successive speciation events, followed by reciprocal allele fixation in each of the descendant lineages, i.e., ILS. We define the extent of ILS as corresponding to weak conflict (persistence across two speciation events; Fig 1C), moderate conflict (three events; Fig 1D), or strong conflict (more than three events; Fig 1E). Per-branch counts of ILS-affected RE insertion events show that incongruences are pronounced on some internodes and are nearly absent on others (Fig 1A and 1B), with greater conflict in deeper internodes. The internodes among core waterbirds exhibit weaker discordances and large amounts of conflict-free RE markers, which is in line with the observation that the RE relationships are fully congruent with the genome-level sequence analyses in Jarvis et al. [4]. Within core landbirds, the MPRE tree is fully congruent with a genome-level tree based on sequences from UCEs [4], yet discordant with the main tree from Jarvis et al. [4] with regards to the position of mousebirds. The deepest divergences of core landbirds contain many ILS-affected markers with strong discordances when mapped on the main Jarvis et al. tree [4] (Fig 1A), but slightly less so when mapped on the MPRE topology (Fig 1B). Furthermore, the placement of owls in the MPRE tree (Fig 1B) is in agreement with our preliminary analysis of owl REs [4]. However, we emphasize that an alternative grouping of owls with eagles and New World vultures received nearly as strong RE support [4], which suggests that the position of the owls may in fact approximate a trifurcation. Among the remaining neoavian divergences, nine internodes are discordant between our MPRE tree and the genome-scale sequence tree (Fig 1A and 1B), all of which are characterized by scarcity of ILS-free RE markers and dominance of RE presence/absence patterns that show complex incongruences resulting from ILS across at least four consecutive speciation events (sensu Fig 1E).

### The Mousebird Conflict Is Not Caused by Hybridization

**Fig 2:**
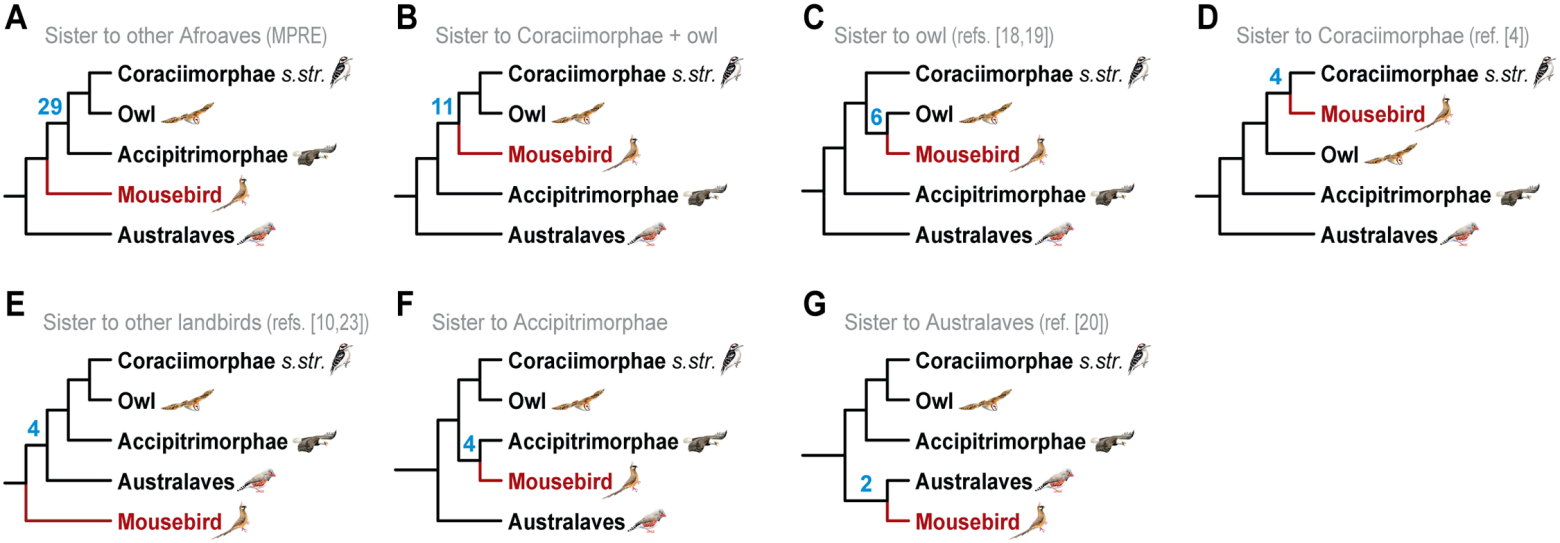
Comparison of RE marker support for the possible positions of mousebirds within core landbirds. The seven alternative groupings are shown in descending order of support and include the mousebird topology of (A), our MPRE tree and the genome-level UCE tree of [4], (C) the Hackett et al. tree [18], (D) the main Jarvis et al. tree [4], (E) two limited retrotransposon studies [10,23], and (G) the main McCormack et al. tree [20]. Blue bold numbers indicate the amount of RE insertion events that are conflict-free with each of the seven alternatives, respectively. Higher-level taxon names are shown for well-supported monophyla, such as the eagles/New World vulture clade (Accipitrimorphae [4]), the passerine/parrot/falcon/seriema clade (Australaves [25]), and the woodpecker/bee-eater/hornbill/trogon/cuckoo-roller clade (Coraciimorphae [4], sensu stricto without mousebird). The bird paintings were generated by Jon Fjeldså (used with permission).

It is striking that the conflicting placements of mousebirds are well-supported in the main Jarvis et al. tree [4] on the one side and our MPRE tree and the genome-scale UCE tree [4] on the other side, respectively. One explanation for this could be hybridization of two distinct and diverged ancestral species, e.g., the ancestor of mousebirds and the ancestor of the woodpecker/bee-eater/hornbill/trogon/cuckoo-roller clade, which would lead to well-supported alternative topologies with conflicts across multiple well-supported branches. This form of hybridization would then be distinguishable from ILS by an over-representation of RE markers supporting one alternative, species tree-incongruent topology and an under-representation of markers supporting the remaining alternative topologies. Such a situation was recently suggested for the very base of the rodent phylogeny [22]. We therefore analyzed all six possible positions of mousebirds within Afroaves (core landbirds without the passerine/parrot/falcon/seriema clade) for their respective support by RE markers. We also analyzed RE support for a grouping of mousebirds as sister to the remaining core landbirds, which was previously suggested in limited RE studies that used the zebra finch genome as only query species [10,23]. The strongest support (29 RE markers) was found for mousebirds as the sister taxon of the remaining Afroaves (Fig 2A), and the six alternatives were recovered by two to eleven markers each (Fig 2B–2G), with four markers supporting the Jarvis et al. topology of mousebirds being sister to Coraciimorphae *s*. *str*. [4] (Fig 2D). The fact that we found no excess of markers supporting the main Jarvis et al. topology [4] over the other alternatives suggests that the mousebird conflict was not caused by hybridization. Instead, the nearly symmetric distribution of support among the six non-MPRE topologies indicates that the presence/absence patterns of these RE markers result from stochastic sorting of alleles after persistence of ILS across the early diversification of core landbirds. We thus suggest that the whole-genome and intron-specific sequence trees [4] recover a locally anomalous topology [15] driven by the known problematic behavior of mousebirds in sequence analyses [24]. We emphasize that the genome-scale UCE tree [4] supports exactly the same mousebird affinities as the majority of REs herein (Figs 1B and 2A), raising the question as to how the UCE phylogenetic signal was overruled by intronic signal within the main genome-scale sequence analysis of Jarvis et al. [4].

### Temporal Extent of Incomplete Lineage Sorting

**Fig 3:**
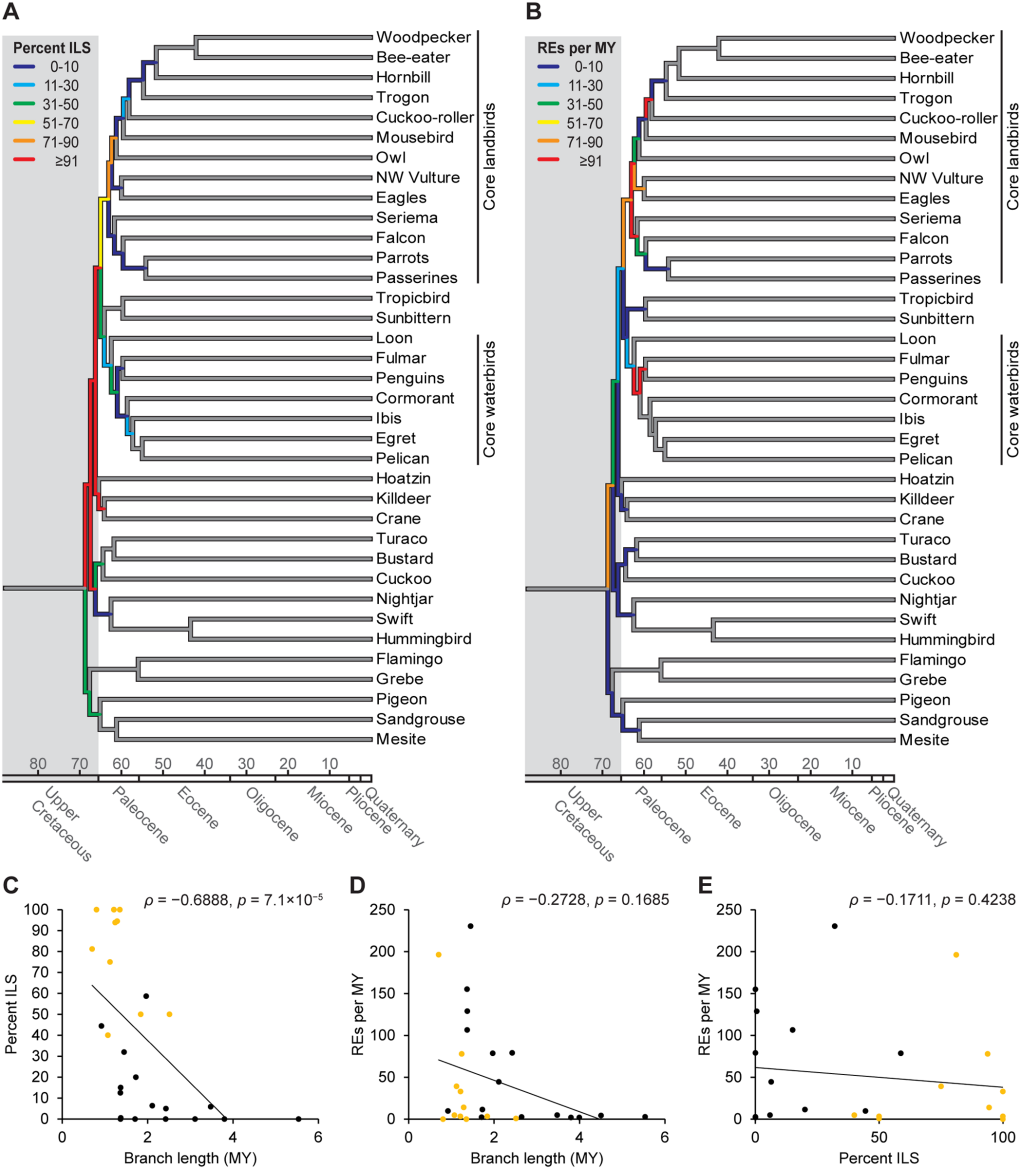
Dynamics of incomplete lineage sorting and RE insertion rates across the dated main Jarvis et al. tree [4]. Per-branch levels of ILS (A) and RE insertion rates (B) vary considerably across the diversification of Neoaves. We derived these values from mapping our 2,118 RE markers on the main Jarvis et al. tree [4] (Fig 1A). For each branch, percentages of ILS were calculated by dividing the amount of ILS-affected markers by the total amount of markers (S2 Table). The latter value was then divided by the respective branch length to estimate the RE insertion rate per MY (S2 Table). Notably, branch length and degree of ILS correlate negatively (S2 Table) (C), but there is no correlation between branch length and RE insertion rate (S2 Table) (D) or between degree of ILS and RE insertion rate (S2 Table) (E). Orange dots denote those branches that are incongruent between the main Jarvis et al. tree [4] and our MPRE tree (cf. Fig 1A and 1B).

Mapping RE markers on a dated time tree of the main Jarvis et al. analysis [4] enabled us to estimate the temporal dynamics of ILS across the very short internodes of the neoavian radiation (Fig 3A). Jarvis et al. infer the onset of Neoaves diversification at around the K-Pg boundary [4], which is in stark contrast to most mitochondrial and multilocus nuclear studies (but see refs. [17,26]) that estimate the deepest neoavian divergences at >82 million years ago (MYA) [3,27] or even >100 MYA (reviewed by ref. [28]). We anticipate that this debate will persist for the upcoming years. However, given that the Jarvis et al. [4] estimates are the first based on genome-scale data, we consider these to be the most reliable molecular dates currently available. We found a negative correlation between branch length and the percentage of ILS-affected RE markers per branch (Spearman's *ρ* = −0.6888, *p* = 7.1×10^−5^; Fig 3C), which corroborates our assumption that ILS is indeed the driving force for most (if not all) of the observed incongruences. This is due to the fact that ILS has a higher probability of occurring if the time between consecutive speciation events is short [29,30], and we would expect no such correlation if the conflicts we refer to as ILS-derived were instead caused by homoplasy. Strikingly, the per-branch estimates of ILS (Fig 3A) suggest that all those branches (or their 95% credible interval of divergence times in ref. [4]) overlapping with the K-Pg boundary exhibit 40%–100% ILS and are mostly incongruent with our MPRE tree (Figs 1B and 3C). Furthermore, the three deepest branches within the post-K-Pg diversification of core landbirds are affected by 59%–81% ILS, including the two branches involved in the aforementioned mousebird conflict. This means that with the exception of the core waterbird/sunbittern/tropicbird branch and the core landbird branch, all branches affected by ≥40% ILS were incongruent with our MPRE tree (Figs 1B and 3C).

We then tested if temporal variation in RE insertion rates (Fig 3B) may account for some of the irresolution. While there is considerable rate variation between branches (Fig 3B), there is no correlation between branch length and RE insertion rates (Fig 3D) or between RE insertion rates and degree of ILS (Fig 3E). Altogether, this suggests that the low amount of ILS-free markers on the problematic branches is not the result of very low RE insertion rates (<10 REs per MY). This is further supported by the notion that most of the post-K-Pg branches within core landbirds and core waterbirds have similarly short branch lengths and mostly low RE insertion rates, yet also low ILS (e.g., core waterbird branch with 12 RE insertions per MY and 20% ILS). We thus propose that the high prevalence of ILS ranging from 40%–100% across the deepest relationships among Neoaves is not an issue of taxon or marker sample size and rather reflects the biology of very rapid speciation.

### Phylogenetic Extent of Incomplete Lineage Sorting

**Fig 4:**
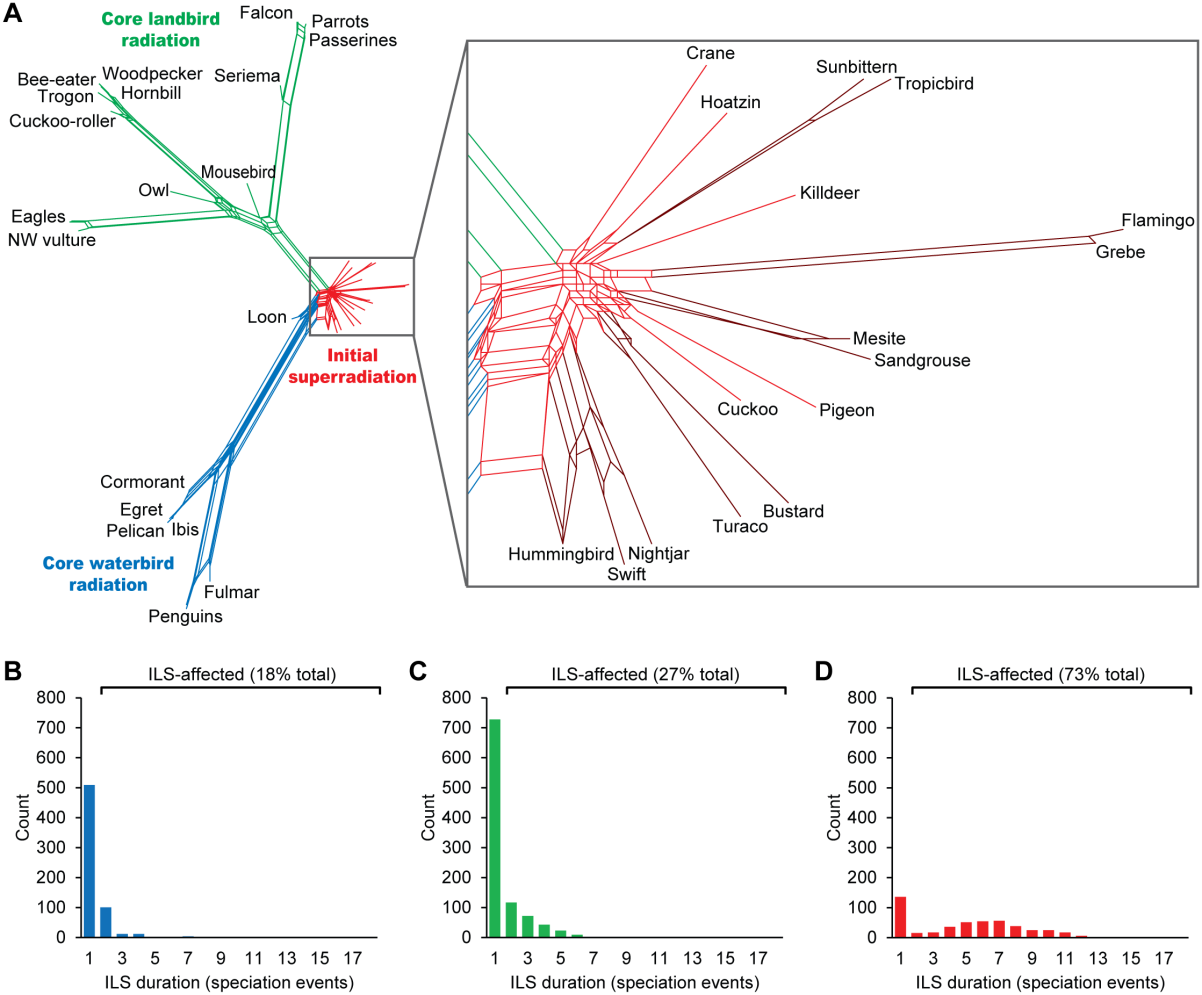
Phylogenetic network of rare genomic changes reveals three adaptive radiations of Neoaves with varying complexity of genealogical incongruences. (A) Neighbor-net [31] analysis of 2,118 RE presence/absence patterns suggests that Neoaves diversification may be more accurately visualized as a largely bifurcating tree with highly reticulate structures at the base of the core landbird radiation and across most of the initial super-radiation. Within the latter, red-brown reticulations highlight bifurcate relationships (cf. Fig 1A and 1B) with limited conflict if stretched boxes are longer than they are wide. In contrast, the core waterbird radiation exhibits limited conflict and appears fully bifurcating (cf. Fig 1A and 1B). (B–D) Distribution of frequencies of RE markers without and with ILS (i.e., persistence across ≥two speciation events) for each of the three adaptive radiations (S3 Table). (B) Core waterbird radiation with 18% total ILS, mostly across two speciation events. (C) Core landbird radiation with 27% total ILS, most of which led to weak or moderate conflict via ILS across two to three speciation events. (D) The initial super-radiation exhibits 73% total ILS, almost exclusively with strong discordances caused by persistence of ILS across five or more speciation events.

Complex gene tree–species tree incongruences of retrotransposon markers might be more accurately represented in phylogenetic networks where data conflicts are evident as reticulate relationships among taxa and alternative topologies are visible even within well-supported lineages [12,29]. The resultant neighbor-net [31] (Fig 4A) illustrates the differential distribution of ILS-derived incongruences across the neoavian radiation, with well-supported, low-conflict branches leading to the core landbird and core waterbird clades, respectively. Together with the relatively large amount of ILS of polymorphisms originating deep within each of these two clades, and at the very base of Neoaves (Figs 1A–1B and 3A), this conclusively reveals that neoavian evolution went through three adaptive radiations [4]. Notably, the differences in the extent of ILS among these radiations imply that the tempo or demography of speciation may have varied considerably under the circumstances of accelerated diversification. More precisely, most of the 18% ILS-affected RE insertion events within the core waterbird radiation did not sort completely across two speciation events (Fig 4B), whereas 27% of the insertions in the core landbird radiation did not sort across mostly two to three speciation events (Fig 4C). These percentages of total ILS are comparable to the 34% genome-wide ILS found among human/chimpanzee/gorilla gene trees [7]. Finally, the deepest radiation of Neoaves exhibits discordances in 73% of the RE markers, mostly explained by persistence of ILS across five to seven speciation events (Fig 4D). This is consistent with a highly reticulate network structure (Fig 4A), which is restricted to those internodes that overlap with the K-Pg transition (Fig 2A). Notably, only these neoavian relationships remained unresolvable in whole-genome sequence analyses [4], and ILS-free RE insertions are scarce on these internodes (Figs 1A–1B and 3A).

### Genomic Extent of Incomplete Lineage Sorting

**Fig 5:**
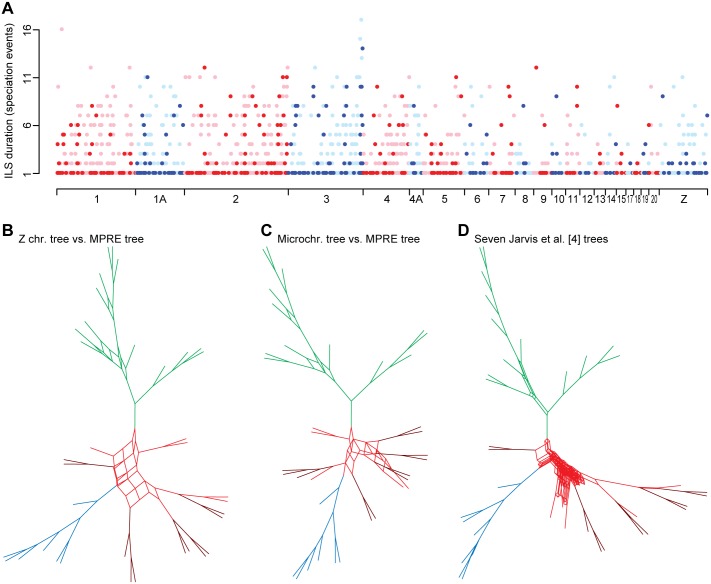
Genomic location of rare genomic changes reveals prevalence of incomplete lineage sorting across all chromosomes. (A) Distribution of RE markers across the chromosomes of the zebra finch genome plotted against per-marker count of speciation events during which respective ILS persisted (S1 Table). Locations of intronic markers are indicated in dark colors, while putatively intergenic markers are denoted in light colors. (B–D) Supernetworks [31] illustrate the complex reticulations of topology conflicts between compared trees. These conflicts exhibit highly similar distributions (cf. Fig 4A) in supernetwork comparisons between the MPRE tree (Fig 1B, S2 Data) and a tree based on 114 Z-chromosomal REs (S2 Data) (B), between the MPRE tree and a tree based on 140 REs from microchromosomes (i.e., all chromosomes smaller than 20 Mb; S2 Data) (C), and among seven different genome-level sequence analyses from Jarvis et al. [4] (D). Colors of reticulations correspond to the coloration used in Fig 4 for discerning the three adaptive radiations of Neoaves. See Supporting Information (S3 and S4 Figs) for species labels and details on the trees compared in supernetworks.

Retrotransposon loci affected by ILS are distributed across the avian genome, irrespective of the duration of ILS per intronic or intergenic marker (Fig 5A). This situation likely applies to the ancestral Neoaves genome, as the avian karyotype is unusually stable, including conserved synteny of the Z sex chromosome and ubiquitous presence of numerous microchromosomes [32,33]. The Z chromosome (no recombination in female meiosis) represents a low recombination environment [34–37] and is affected by ILS to a similar extent (34%) as the genome-wide average of 35% (S2 Table), and a tree based on RE markers from the Z chromosome yields similar topological discordances with the MPRE tree (Fig 5B and 5C, S3 Fig). This is contrary to the observation of previous studies (on nonrapid diversifications), where low-recombination autosomal regions and sex chromosomes generally exhibit less ILS due to the lower effective population size (*N*_*e*_) of regions with low recombination [7,30]. Finally, it is striking that the incongruences among our chromosomal trees of rare genomic changes almost perfectly overlap with conflicts among whole-genome sequence trees derived from concatenated or coalescence-based analyses of various data partitions in Jarvis et al. [4] (Fig 5D, S4 Fig) and again yield a highly reticulate structure at the base of Neoaves (cf. Fig 4A). Taken together, this reveals that many of these deepest neoavian divergences receive considerable support in some and strong refutation in other analyses, suggesting that the consecutive arrangement of their very short internodes may potentially represent Rosenberg’s “anomaly zone” [38], i.e., observing stronger support for a gene tree than for the actual species tree [15].

### Complexity of the Neoavian Radiation

**Fig 6:**
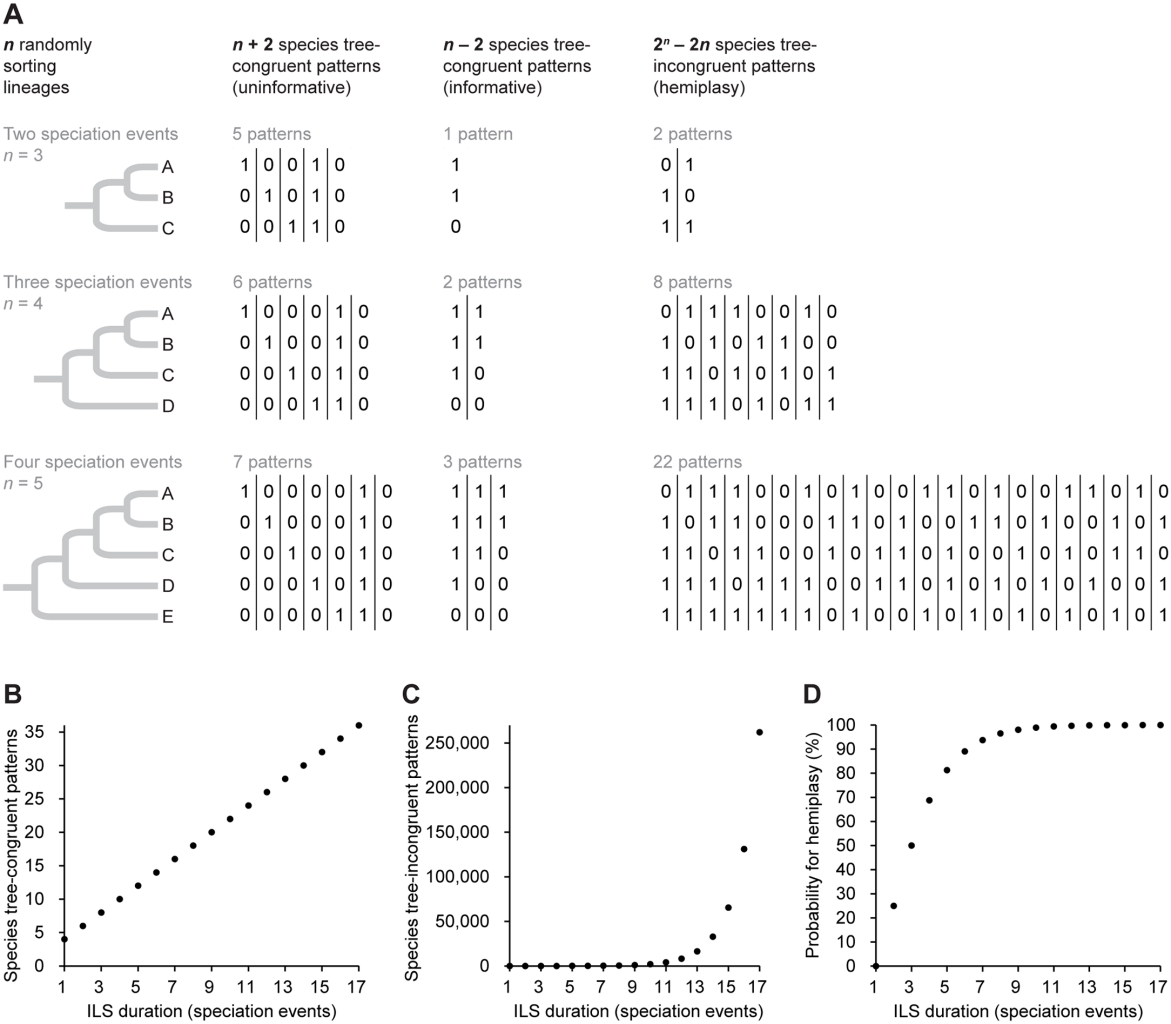
Longer ILS duration of a biallelic polymorphism leads to an exponential increase of hemiplasy. (A) Illustration of all the RE presence/absence patterns that are theoretically possible after ILS across two to four speciation events (extension of the examples shown in Fig 1C–1E). This permitted us to calculate the amounts of possible character distributions that are incongruent or congruent with the species tree under the observed durations of ILS across up to 17 speciation events (S5 Table). Note that conflict-free patterns parsimoniously correspond to ILS across one or fewer speciation events. (B) The amount of species tree-congruent patterns increases linearly (2*n*) with ILS duration. (C) The amount of hemiplasy (i.e., species tree-incongruent patterns) increases exponentially (2^*n*^ *–* 2*n*) with ILS duration. (D) The probability for the occurrence of hemiplasy in a biallelic polymorphism reaches 50% after ILS across three speciation events and 99% after ILS across eleven speciation events (S5 Table).

The probability for the occurrence of ILS depends on *N*_*e*_ in relation to the time between consecutive speciation events [29,30], with *N*_*e*_ correlating positively and time negatively with the expected extent of ILS, respectively. The observed complex genealogical fates of ancestral RE insertion polymorphisms during the initial super-radiation (Fig 4D) therefore suggest that the onset of neoavian diversification was characterized by a large number of near-simultaneous speciation events of an ancestral species with large *N*_*e*_. Considering the sheer amount of differing allelic combinations that are possible to result from stochastic sorting of ancestral biallelic genetic variation after up to 17 speciation events (Fig 4D), we hypothesize that such complex signals might overrule the underlying species tree-concordant signal, because the latter can be expected to occur rarely under the complex sorting scenario envisioned (cf. Figs 1A–1B and 3A). Considering all theoretically possible RE presence/absence patterns in a five-taxon tree (Fig 1C–1E), ILS across four speciation events requires allelic sorting in each of the descendant lineages, permitting 22 different character distributions that are discordant with the species tree (Fig 6A, S5 Table). Under the model of stochastic sorting of polymorphisms of RE presence/absence (Fig 6) or other types of biallelic variation (e.g., single nucleotides), the probability for the occurrence of hemiplasy surpasses 90% after an ILS duration of seven speciation events (Fig 6D, S5 Table). This may explain why the deepest neoavian bifurcations receive various alternative topologies in the different genome-scale sequence trees of Jarvis et al. [4] (Fig 5D). However, the high bootstrap support (>90%) for some alternative bifurcations could also mean that there are several comparably likely relationships, thus resembling a local network. Alternatively, Salichos & Rokas recently proposed that bootstrapping in phylogenomic analyses can lead to strong support for bifurcations even in the light of strong conflict [39]. Even if one of these genome-scale bifurcating trees reflects the actual neoavian species tree, the verification of such a phylogenetic hypothesis remains challenged by the underlying complex discordances. Finally, the nearly star-shaped topology of this super-radiation (Figs 4A and 5D) may reflect population complexity of the ancestral species, especially if the succession of population isolation during explosive speciation happened in disagreement with prior population structure [40,41].

### Conclusions

We conclude that Neoaves diversification is more complex than can be shown in fully bifurcating trees and exhibits a dynamic picture of ILS. The timing of the highly ILS-affected initial super-radiation coincides with the K-Pg extinction of nonavian dinosaurs and archaic birds [42], suggesting that the abrupt availability of ecological niches [4] was followed by near-simultaneous population isolations [41] via specializations and led to several network-like relationships. The subsequent, decelerated adaptive radiations of waterbirds and raptorial [4] landbirds exhibit less ILS and likely took place after the K-Pg boundary [4,17]. Interestingly, this time span is similar to placental mammal diversification [43], which was accompanied by localized and less pronounced ILS than shown here for Neoaves [12,13]. Finally, and contrary to the expectation that complete genomes will permit full resolution of phylogenies [44], our genome-level analyses of rare genomic changes yield a broadly bifurcating species tree of Neoaves [4] with local network-like reticulations that probably lie in the anomaly zone. Our study thus provides empirical evidence for a locally confined “hard” polytomy [41], and we predict that future genome-wide studies of ILS in other adaptive radiations will reveal further examples where a fully bifurcating, universal species tree is an oversimplification of the underlying complexity of speciation.

## Materials and Methods

### Taxon Sampling

Our taxon sampling comprises the genome assemblies of 48 recently sequenced birds [45] and thus contains the same species that were used in the genome-scale sequence analyses of Jarvis et al. [4]. We focused on identifying RE insertion events during early neoavian evolution and therefore excluded non-neoavian genomes (chicken, duck, ostrich, tinamou, turkey) from the set of query species used for extracting RE candidate loci. All neoavian genomes were utilized as queries, with the exception of close relatives of ingroup species (as they do not add much more information) such as zebra finch (i.e., four remaining passerines), white-tailed eagle (i.e., bald eagle), budgerigar (i.e., kea), and adelie penguin (i.e., emperor penguin). This taxon sampling contains representatives of all major neoavian lineages [4], and we thus consider it sufficient for estimating ILS during Neoaves diversification. We expect that the addition of more taxa via sequencing of additional genomes would not result in an improved resolution of our RE data but rather lead to an increase of missing data and the detection of additional ILS across internodes that lie outside the three neoavian radiations reported herein.

### Retrotransposon Screening

We analyzed a total of ~130,000 copies of *hitchcock*-related LTR retrotransposons [46] that were previously shown to be REs characteristic of early bird evolution [10,47] and constitute the majority of RE activity during the neoavian radiation [10]. After repeat annotation of the sampled genomes using RepeatMasker [48] version 3.2.9, we extracted all TguLTR5d elements for each query species, including 1-kb flanks per RE locus. These sequences were then compared to the remaining query species via BLASTn [49] (cutoff *E* < 10^−30^), followed by extraction of the BLASTn hits and generation of locus-specific alignments in MAFFT [50] (version 6, E-INS-i). These alignments were postfiltered to exclude loci exhibiting less than ten species, missing flanks, plesiomorphic RE insertions (i.e., orthologous presence among all query species), or autopomorphic RE insertions (i.e., presence only in one query species). Furthermore, we omitted loci that were redundant or potentially paralogous. Among the ~8,000 remaining candidate loci, we manually identified phylogenetically informative RE insertions (including additional RE insertions in the sequences flanking the TguLTR5d query) in ~3,000 loci. For these marker candidates, we compiled final multispecies alignments after BLASTn searches (cutoff *E* < 10^−10^) of 2-kb flanks against the full taxon sampling of 48 birds.

### Presence/Absence Analysis

RE markers serve as virtually homoplasy-free estimators of ILS-derived hemiplasy after minimizing potential errors that might arise from misalignment or incorrect scoring. Therefore, we carefully inspected the 48-species presence/absence alignment of each of the ~3,000 marker candidates by eye and manually coded binary character states using strict standard criteria [10,51,52]. Character state “1” requires the presence of an orthologous RE insertion (i.e., identical insertion point, orientation, RE subtype, and target site duplication) in an orthologous genomic locus (i.e., single-copy flank regions). Character state “0” constitutes the absence state of a particular RE insertion, as indicated by the presence of a nonduplicated target site and an alignment gap precisely corresponding to the RE presence/absence boundaries. If neither of the conditions necessary for character states “1” or “0” were met, character states were treated as missing data and coded as “?”. The same was done in the case of a gap in the genome assembly or a large unspecific deletion of the insertion locus. Marker candidates that did not meet the aforementioned strict criteria were omitted. This overall procedure led to a reduction of the ~3,000 marker candidates to a final set of 2,118 RE markers. Note that from these markers, 61 were previously published as a preliminary analysis of owl retrotransposons with the focus on determining the owl sister group [4,53]. We emphasize that these 2,118 markers encompass all RE insertion events during the neoavian radiation that were identified with our screening approach, with the exception of shallower internodes because they were not the main focus of our analyses of neoavian ILS. In the latter cases, we recorded a subset of the numerous marker candidates for these internodes, namely the woodpecker/bee-eater/hornbill, hummingbird/swift, pelican/egret/ibis/cormorant, and flamingo/grebe clades, as well as internodes within these.

### Target Site Analysis

We manually recorded target site duplications (i.e., direct repeats of 5 bp flanking the studied LTR retrotransposons) for each of our RE markers (S1 Table). This was done by visually inspecting the left and right flanks in our 48-species marker alignments to parsimoniously infer the putative ancestral states of lineage-specific nucleotide changes in each orthologous target site. We therefore suggest that these reconstructed motifs approximate the respective target sequences at the time points of RE insertion. We analyzed the motifs in 5′–3′ orientation (relative to the LTR orientation) using WebLogo [54]. The resultant sequence logo [55] contains near-equal frequencies of the four possible nucleotides per motif position (S2 Fig), which suggests that there is no target site preference among the REs studied herein.

### Phylogenetic Analysis

We analyzed the 1/0-coded presence/absence matrix of 2,118 RE markers using the Dollop program in PHYLIP [56] version 3.695 under polymorphism parsimony and standard parameters with randomized input order of species (7 times to jumble, random seed “11111”). The Dollop output contained the resultant MPRE tree and the parsimony-inferred per-branch character states for each RE marker, which we used to calculate the amount of ILS-free markers per internode, and to infer the duration of ILS across speciation events in incongruent insertions. We also ran Dollop using the main Jarvis et al. tree [4,53] as input tree under the same aforementioned parameters, which was followed by estimation of the amount and duration of ILS across internodes. Subsequently, *Z*-chromosomal and microchromosomal RE trees were generated using Dollop under the same parameters as the MPRE tree. Finally, Splitstree [31] version 4.13.1 was used for neighbor-net analysis of conflict within our RE presence/absence matrix and supernetwork analyses of conflict between different tree topologies based on REs (S2 Data) or nucleotide sequences [4,53].

### ILS Analysis

Our phylogenetic analyses yielded a reconstruction of transitions of character states for each RE marker, thus allowing the analysis of ILS-derived hemiplasy under the assumed negligibility of homoplasy. We defined an ILS-free marker (i.e., duration of ILS across maximally one speciation event) as one that required a single step when mapped on the analyzed tree. In the Dollop output, this is coded as a single transition to the presence state (“1”) on the branch where the RE insertion occurred. If a presence/absence pattern required more than one step when mapped on the given tree, it was defined as an ILS-affected marker (i.e., duration of ILS across minimally two speciation events). Under polymorphism parsimony, such a pattern results from a polymorphic RE insertion (“P”) that occurred on a branch prior to the conflicting branches and then persisted as a polymorphism across two or more speciation events, followed by stochastic allele sorting in the descendant lineages. We thus counted the total number of transitions to the presence (“1”) and the absence (“0”) allele necessary to explain the given topological conflict as a measure for the minimal amount of independent allele fixation events. Considering that *n* speciation events give rise to *n +* 1 lineages (Fig 6), our estimates of the duration of ILS correspond to the minimum of speciation events across which ILS persisted when counting the minimal amount of lineages that must have independently sorted under the given RE presence/absence pattern. Finally, we manually counted all possible allelic fates for ILS across two to four speciation events (Fig 6A) and derived a formula to calculate the amount of species tree-incongruent presence/absence patterns theoretically resulting from any duration of ILS (Fig 6, S5 Table). Dividing this number of hemiplasious character distributions by the amount of all theoretically possible presence/absence patterns yielded the probability of occurrence of hemiplasy in a biallelic polymorphism (Fig 6C, S5 Table).

## Supporting Information

S1 Data Marker locus sequences in fasta format. Marker names are derived from the first four letters of the scientific name of the query species. Only the sequence from the respective query species is included.
(TXT)

S2 Data Tree files of the MPRE tree (A), the *Z*-chromosomal RE tree (B), and the microchromosomal RE tree (C). Marker names are derived from the first four letters of the scientific name of the query species.
(TXT)

S1 Fig Presence/absence alignments of exemplary ILS-free and ILS-affected markers. (A) The ILS-free marker lept_02654 contains a TguLTR5d retrotransposon insertion (lowercase letters) flanked by a 5′-ATATG-3′ target site duplication (boxed). (B) The ILS-affected marker lept_01115 contains a TguLTR5d retrotransposon insertion flanked by a 5′-TCACC-3′ target site duplication. Following RE insertion (colored circles), the two markers underwent different genealogical fates with the segregation of presence (colored lines) or absence (black lines) alleles prior to a speciation event (A) or after three successive speciation events (B). Six of the 48 species from the original presence/absence alignment are shown, respectively, and the remaining species exhibit the same character state (RE absence) as the owl.
(TIF)

S2 Fig Sequence logo of ancestral sequences of target site motifs reveals no target site preference in the analyzed retrotransposon markers. Ancestral sequences of target site motifs were reconstructed by visually inspecting the 48-species alignment of each RE marker. The height of each nucleotide visualizes its relative frequency at each position of the 5-bp target site motif of the analyzed LTR retrotransposons. This lack of target site preference implies that homoplasy arising from independent insertion of the same RE into the same orthologous target site is negligible.
(TIF)

S3 Fig Supernetworks of retrotransposon trees with taxon labels and tree information. (A) Supernetwork of MPRE tree and Z-chromosomal RE tree (cf. Fig 5B; S2 Data). (B) Supernetwork of MPRE tree and microchromosomal RE tree (cf. Fig 5B; S2 Data). Colors of reticulations correspond to the coloration used in Fig 4 for discerning the three adaptive radiations of Neoaves.
(TIF)

S4 Fig Supernetwork of seven genome-scale sequence analyses from Jarvis et al. [4] (cf. Fig 5D). Concatenated and coalescence-based analyses are indicated and involve introns, indels (TEIT), exons/introns/UCEs (TENT), ultraconserved elements (UCEs), or whole-genome alignments (WGT). Tree files are from Jarvis et al. [4,53], and colors of reticulations correspond to the coloration used in Fig 4 for discerning the three adaptive radiations of Neoaves.
(TIF)

S1 Table Presence/absence matrix of 2,118 retrotransposon markers including RE target site motifs, ILS duration, and location in the taeGut2 assembly of the zebra finch genome. (XLS)

S2 Table Per-branch levels of ILS and RE insertion rates across neoavian internodes of the main tree from Jarvis et al. [4]. (XLS)

S3 Table Distribution of frequencies of ILS-affected RE markers across the three radiations of Neoaves. (XLS)

S4 Table Per-chromosome distributions of retrotransposon markers including levels of ILS, marker density, and exon density in the taeGut2 assembly of the zebra finch genome. (XLS)

S5 Table Expected probability for hemiplasy following random fixation of a biallelic polymorphism under incomplete lineage sorting across 1–17 speciation events. (XLS)

## Abbreviations

ILS: incomplete lineage sorting
K-Pg: Cretaceous-Paleogene
LTR: long terminal repeat element
MPRE: most parsimonious RE
MY: million years
MYA: million years ago
*N*_*e*_: effective population size
RE: retrotransposed element
UCE: ultraconserved element
