# Supplementary figures and images for: The Dynamics of Incomplete Lineage Sorting across the Ancient Adaptive Radiation of Neoavian Birds
Source: PLoS Biol. 2015 Aug 18;13(8):e1002224. doi: 10.1371/journal.pbio.1002224 (PMC4540587; doi:10.1371/journal.pbio.1002224)

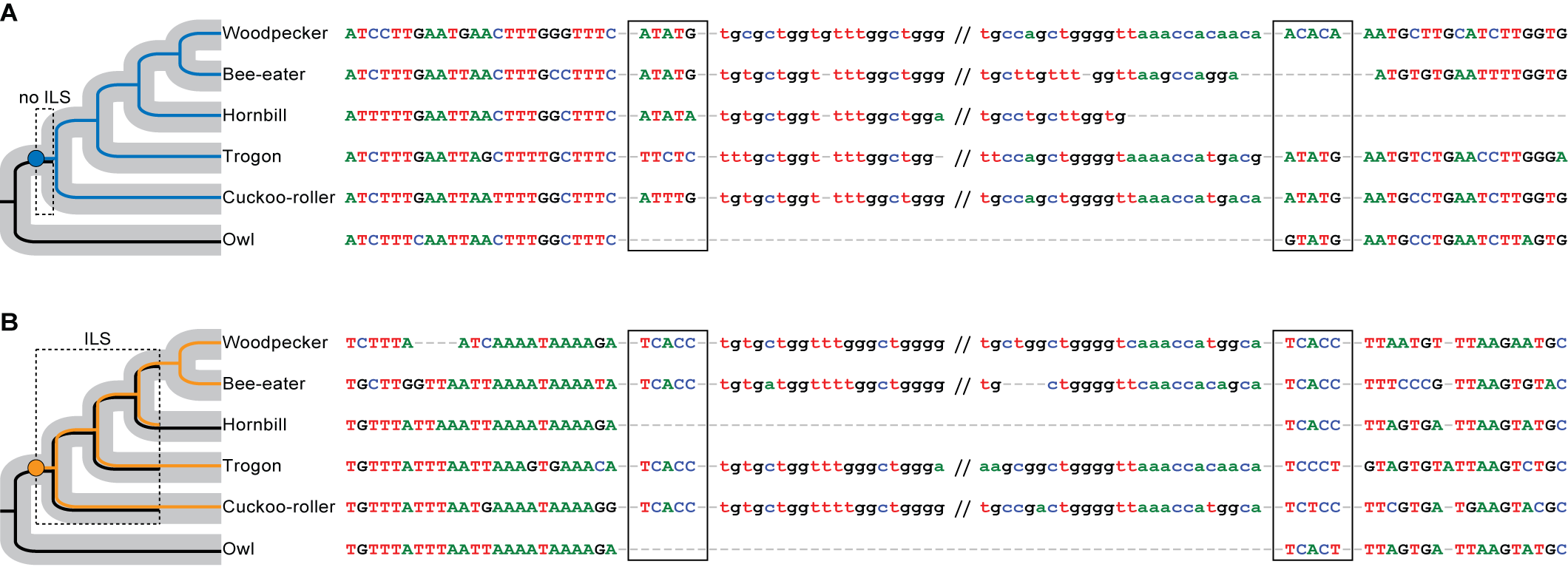

Supplement: S1 Fig — (A) The ILS-free marker lept_02654 contains a TguLTR5d retrotransposon insertion (lowercase letters) flanked by a 5′-ATATG-3′ target site duplication (boxed). (B) The ILS-affected marker lept_01115 contains a TguLTR5d retrotransposon insertion flanked by a 5′-TCACC-3′ target site duplication. Following RE insertion (colored circles), the two markers underwent different genealogical fates with the segregation of presence (colored lines) or absence (black lines) alleles prior to a speciation event (A) or after three successive speciation events (B). Six of the 48 species from the original presence/absence alignment are shown, respectively, and the remaining species exhibit the same character state (RE absence) as the owl. (TIF) [file pbio.1002224.s003.tif]

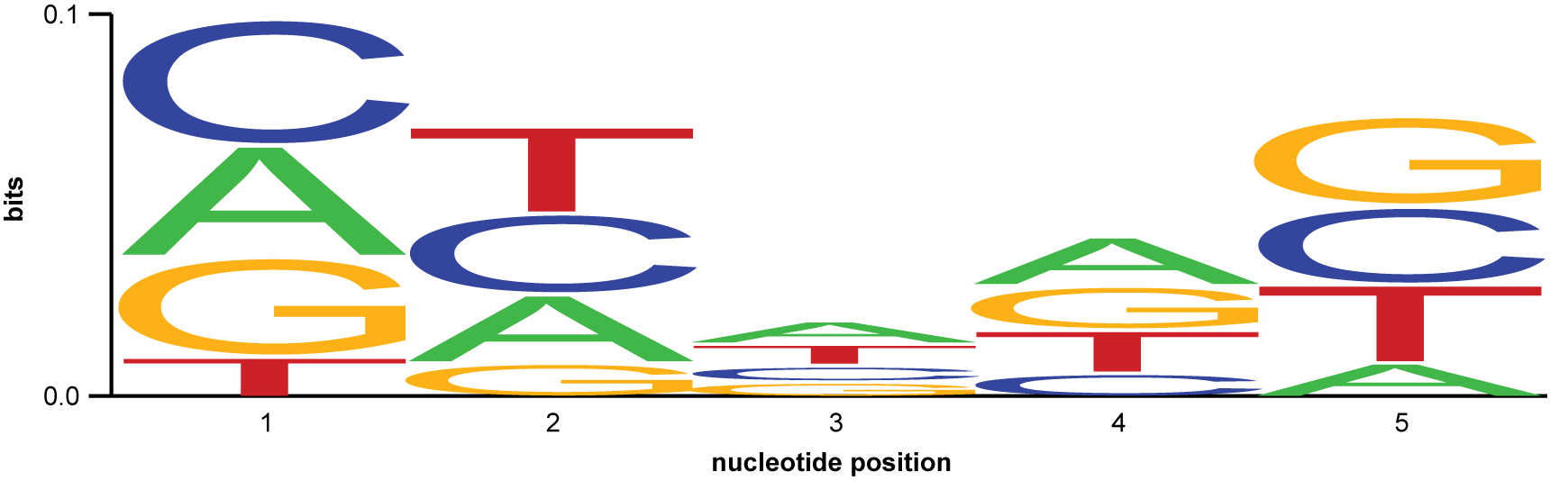

Supplement: S2 Fig — Ancestral sequences of target site motifs were reconstructed by visually inspecting the 48-species alignment of each RE marker. The height of each nucleotide visualizes its relative frequency at each position of the 5-bp target site motif of the analyzed LTR retrotransposons. This lack of target site preference implies that homoplasy arising from independent insertion of the same RE into the same orthologous target site is negligible. (TIF) [file pbio.1002224.s004.tif]

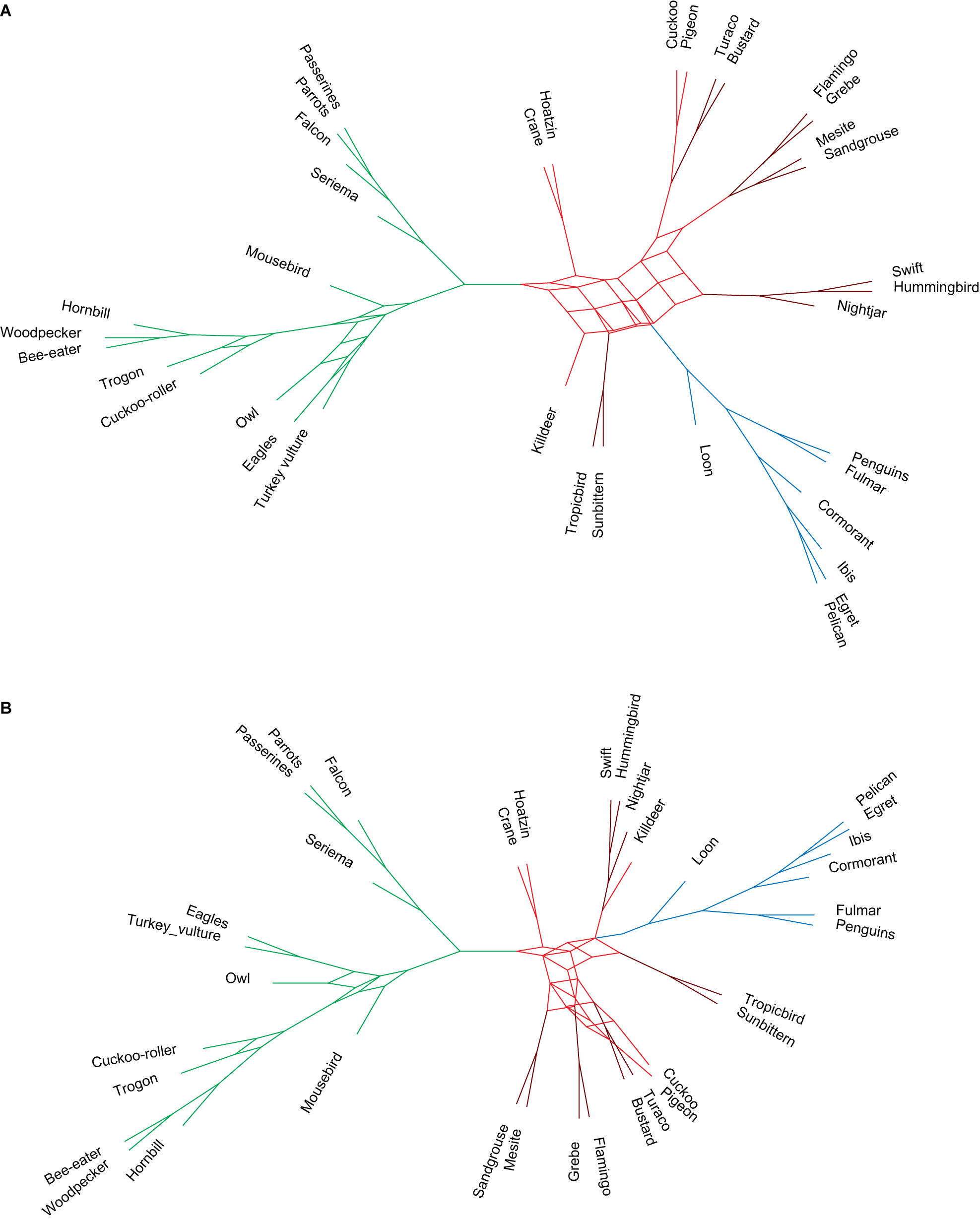

Supplement: S3 Fig — (A) Supernetwork of MPRE tree and Z-chromosomal RE tree (cf. Fig 5B; S2 Data). (B) Supernetwork of MPRE tree and microchromosomal RE tree (cf. Fig 5B; S2 Data). Colors of reticulations correspond to the coloration used in Fig 4 for discerning the three adaptive radiations of Neoaves. (TIF) [file pbio.1002224.s005.tif]

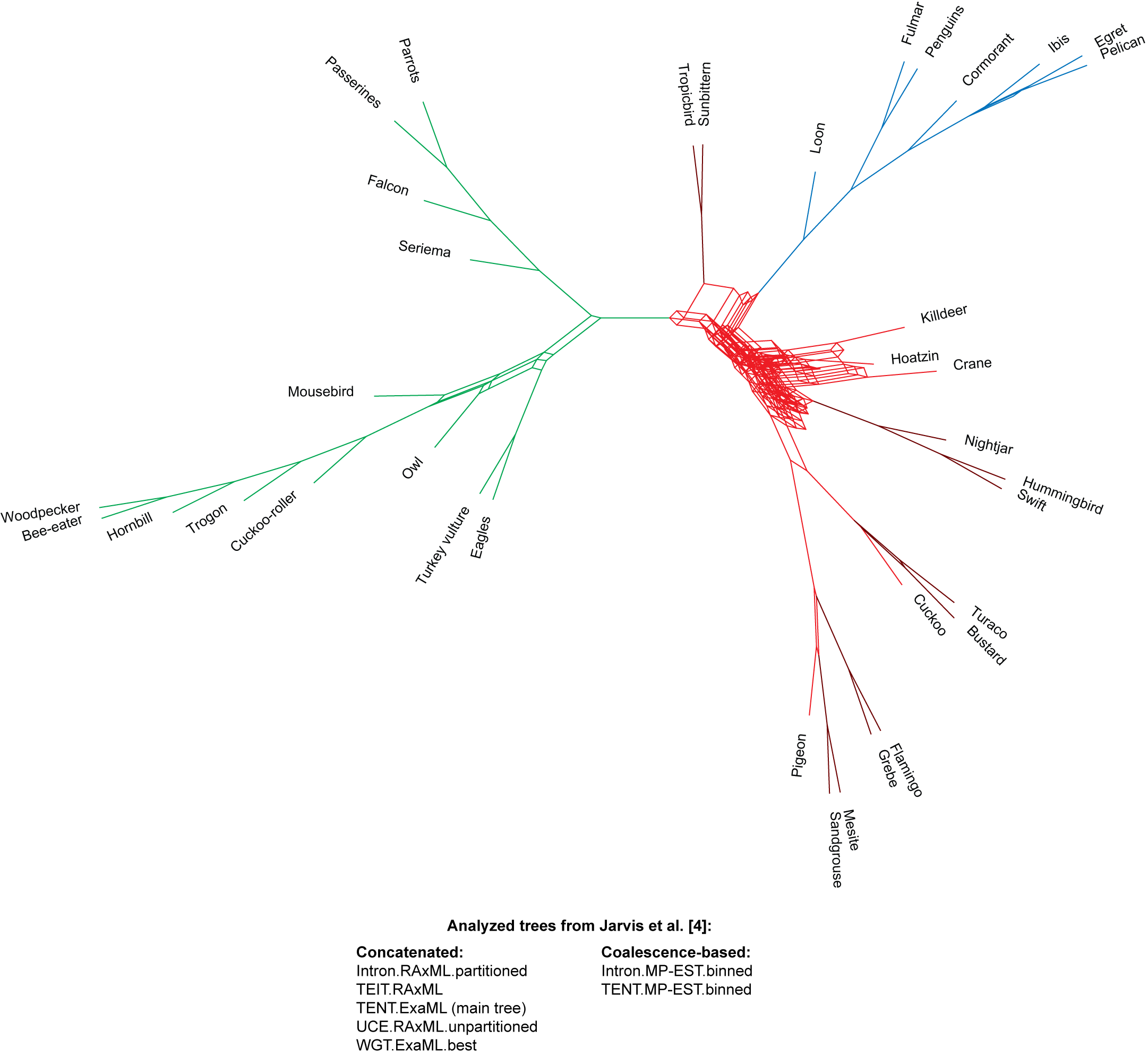

Supplement: S4 Fig — Concatenated and coalescence-based analyses are indicated and involve introns, indels (TEIT), exons/introns/UCEs (TENT), ultraconserved elements (UCEs), or whole-genome alignments (WGT). Tree files are from Jarvis et al. [4,53], and colors of reticulations correspond to the coloration used in Fig 4 for discerning the three adaptive radiations of Neoaves. (TIF) [file pbio.1002224.s006.tif]
